# Effect of a rare genetic variant of *TM7SF4* gene on osteoclasts of patients with Paget’s disease of bone

**DOI:** 10.1186/s12881-017-0495-3

**Published:** 2017-11-16

**Authors:** Emilie Laurier, Nathalie Amiable, Edith Gagnon, Jacques P. Brown, Laëtitia Michou

**Affiliations:** 1 CHU de Québec-Université Laval Research Centre, Quebec, QC Canada; 2 Department of Medicine, Université Laval, Quebec, QC Canada; 3 Department of Rheumatology, CHU de Québec-Université Laval, Quebec, QC Canada; 4 Rhumatologie- R4774, CHU de Québec-Université Laval, 2705 boulevard Laurier, Québec, Québec G1V 4G2 Canada

**Keywords:** Paget’s disease of bone, *TM7SF4* gene, DC-STAMP protein, Rare genetic variant, Osteoclast

## Abstract

**Background:**

Dendritic Cell-Specific Transmembrane Protein (DC-STAMP) is involved in osteoclastogenesis with a key role in mononucleated osteoclasts fusion. We reported in patients with Paget’s disease of bone (PDB) a rare variant (rs62620995) in the *TM7SF4* gene, encoding for DC-STAMP, which changes a highly conserved amino acid, possibly damaging according to in silico predictions. This study aimed at determining the functional effects of this variant on osteoclast phenotype in PDB.

**Methods:**

Fifty ml of peripheral blood were collected in pagetic patients carrier of this variant (*n* = 4) or not (*n* = 4) and healthy controls (*n* = 4). Monocytes were collected after Ficoll gradient and cultured in a medium containing RANKL (40 ng/ml) and hMCSF (25 ng/ml). At the end of the differentiation period, we assessed the osteoclast morphology and bone resorption abilities. We quantified gene expression of *SQSTM1, DC-STAMP, OS9, CREB3, LAMP1, OC-STAMP*, and *NFATC1* genes from cell lysates. Proteins encoded by these genes were investigated by Western Blot. Statistical analyses relied on ANOVA followed by Tukey post-tests.

**Results:**

After 21 days of differentiation, the mean number of nuclei per multinucleated cell was significantly higher in pagetic patients carrier of the variant than in healthy controls. Bone resorption abilities were not modified by the variant. qPCR and Western Blot analyses did not provide any differences, but DC-STAMP expression was higher in patients carrier of the variant than in patients non carrier.

**Conclusions:**

This *TM7SF4* rare variant may have an impact on osteoclast morphology and on DC-STAMP expression during osteoclastogenesis. Further analyses are required to understand the role of this variant during osteoclastogenesis in PDB.

**Electronic supplementary material:**

The online version of this article (10.1186/s12881-017-0495-3) contains supplementary material, which is available to authorized users.

## Background

Paget’s disease of bone (PDB) is a common metabolic bone disorder affecting up to 3% of the Caucasian population after the age of 55 and more than 10% after 80 years of age [1]. This disease is characterized by overactive osteoclasts leading to increased bone resorption followed by a coupled excessive bone formation by osteoblasts [2]. Although every bone may be affected by PDB, vertebrae, skull, pelvis, femur and tibia are the most frequently affected bones. PDB symptoms, which vary depending on the affected bones and the severity of the disease, may go from joint pain to fractures, bone deformity, bone pain, headache, and deafness [3]. The pathophysiology of PDB is not well-understood yet, but environmental, viral and genetic factors are likely to be involved [2, 4]. Fifteen to 30% of patients with PDB have a family history of this disease, which display an autosomal mode of inheritance with high but incomplete penetrance [5–7]. *Sequestosome 1 (SQSTM1)* gene mutations are frequently reported in familial forms of the disease as well as in 10 to 15% of non familial cases [7, 8]. In addition, genome-wide association studies (GWAS) in European patients with PDB identified significant associations of PDB with common genetic variants (SNPs) located in *1p13 (*near *CSF1* gene*)*, in *7q33* (*NUP205* gene), in *8q22* (*TM7SF4*), in *10p13 (OPTN),* in *14q32 (RIN3)* and in *15q24 (PML)* [9–11].

In the French Canadian population, although no allelic association was found with the rs2458413, an intron variant in *TM7SF4* gene (locus *8q22*) previously identified in GWAS, a genotypic association was found for this common variant for the heterozygous genotype *AG* versus both homozygous genotypes *AA* and *GG* (uncorrected *p* = 6.9 × 10^−4^; RR = 1.73 [1.22–2.45]) [12]. Further sequencing candidate genes in novel PDB-associated loci [9–11] allowed our team to identify several rare variants with potential functional effects on PDB phenotypes, in particular a rare variant, rs62620995, located in the fifth exon of the *TM7SF4* gene [12]. In the subgroup of our cohort consisting in pagetic patients non carrier of *SQSTM1* gene mutations, the minor allele frequency of the *T* allele of this variant was twice more frequent in patients with PDB than healthy controls (2.8% versus 1.4%, *p* = 0.09, OR = 2.06 [0.85–5.01]). The global distribution of the rs6262099 variant genotypes differed between patients and controls (*p* = 0.044). 5.6% of patients were heterozygous *CT* versus 2.7% of healthy controls, whereas no homozygous *TT* was found in both groups. The presence of at least one major allele (*C*) in the genotype suggested a protective effect against PDB [12].

The *TM7SF4* gene encodes the DC-STAMP protein (470 amino acids; 53,393 Da), a transmembrane protein involved in osteoclast multinucleation and osteoclastogenesis [13]. The rare genetic variant rs62620995 leads to a non-synonymous change *p. Leu 397 Phe* located in the seventh and last transmembrane domain of the protein. The leucine being highly conserved in the evolution [12] leads us to believe that this amino acid could have an important role in the DC-STAMP protein function. This change was in silico predicted to be damaging (Polyphen, Condel) as it modify an amino-acid located in one of the transmembrane domains of the protein [14], which are critical for this receptor internalization [15].

Since hypermultinucleation is an important characteristic of pagetic osteoclasts, this non synonymous coding rare variant, rs62620995, could contribute to PDB pathophysiology. In this study, we aimed at studying the functional consequences of this variant on osteoclast phenotype and bone resorption abilities, as well as on gene and protein expressions in in vitro differentiated osteoclasts from patients with PDB carriers and non-carriers and healthy controls.

## Methods

### Recruitment of participants

Phenotype assessment of our French Canadian cohort comprised a complete bone evaluation, including total serum alkaline phosphatase, a total body bone scan and skull and pelvis X-rays, as previously reported [12, 16–18]. For all participants, *SQSTM1* gene mutations were searched by Sanger sequencing and rs62620995 was genotyped by Sequenom analysis, as previously reported [7, 12].

### In vitro differentiation of monocytes from the peripheral blood into mature osteoclasts

Fifty ml of peripheral blood were collected in four pagetic patients carrier of the rs62620995 variant, four pagetic patients non carrier of this variant and four healthy controls, all participants being non carrier of any *SQSTM1* mutations. There was no significant difference in terms of age and gender between the three studied groups and participants, nor difference in extent of the disease between the two groups of participants with PDB (Table 1). Lymphocytes and monocytes from the peripheral blood were collected after Ficoll gradient. Adherent cells were cultured in a medium consisting in α-MEM 10% FBS 1% Penstrep (Sigma-Aldrich, Oakville, Can) containing RANKL (Peprotech, Quebec, Can) (40 ng/ml) and hMCSF (Ebioscience, SanDiego, CA) (25 ng/ml). Cells were cultured in Labtek system (Fisher Scientific, Ottawa, Can) and Osteoassay (Fisher Scientific, Ottawa, Can). The medium was changed every two to three days. Fluorescence-based staining for tartrate-resistant acidic phosphatase (TRAP) relying on ELF97 phosphatase substrate (Molecular Probes, Eugene, OR) [19] was performed as well as DAPI (Molecular Probes, Eugene, OR) for nuclei and phalloidin (Molecular Probes, Eugene, OR) for cell membrane. At the end of the differentiation period (21 days), we assessed the osteoclast phenotype by calculating the percentage of multinucleated cells (three nuclei and more) over the total number of cells TRAP positive, the mean number of nuclei per multinucleated cell (among a random sample of 20 multinucleated cells) and the mean area of resorbed bone, by the use of a Nikon Eclipse TE300 fluorescent microscope at 10 X and the ImageJ software.

**Table 1:** Main clinical characteristics of study participants

Status	Mutation	Age range (y)	Age at diagnosis (y)	PAL^a^	Renier’s index (%)	Number of affected bones	Details of involved bones
Healthy	No	60–69					
Healthy	No	50–59					
Healthy	No	70–79					
Healthy	No	70–79					
Affected	CT for rs62620995	40–49	34	1.89	15.70	2	Skull, face excluding the mandibula
Affected	No	70–79	41	10.42	21.35	7	Sacrum, right and left pelvis, left scapula, both humeri, 3 consecutive lumbar vertebrae
Affected	No	80–89	48	5.16	5.00	1	Right tibia
Affected	CT for rs62620995	80–89	64	3.00	27.80	6	Left humerus, D8, D12, right pelvis, and both femurs
Affected	CT for rs62620995	60–69	57	1.24	2.50	1	Left tibia
Affected	CT for rs62620995	75–79	69	1.01	8.20	2	Left proximal femur, left tibia
Affected	No	70–79	74	1.06	6.20	1	2/3 of left femur
Affected	No	50–59	50	1.25	4.50	1	Left pelvis

^a^PAL = total alkaline phosphatase, expressed as the number of time the midpoint of the normal range of total alkaline phosphatase levels

### Immunofluorescence of DC-STAMP protein

Osteoclasts were rinsed with PBS, fixed with paraformaldehyde 4%, rinsed again 3 times for 5 min with PBS and incubated 20 min with PBS 10% FBS. Then they were incubated 60 min with DC-STAMP antibody, 1: 100 in PBS 1.5% FBS (Santa Cruz Biotechnology # sc-87,673, polyclonal rabbit IgG) and rinsed 3 times with PBS. Osteoclasts were also incubated 45 min with anti-rabbit IgG ALEXA Fluor 594, 1: 500 in PBS 1.5% FBS (Cell Signaling 8889S) and rinsed 3 times with PBS. Cells were incubate 3 min with PBS-DAPI (0.3 μM final) (Molecular Probes D1306), rinsed 3 times with PBS and covered with PBS-glycerol 1: 1. Pictures were taken in fluorescence at 40X.

### Quantitative real-time PCR

The total RNA extraction from osteoclast lysates followed by RNA quantification and quality control was performed as described [20]. Quantitative real-time PCR relied on a previously published protocol [21]. Briefly, cDNA was synthesized by the use of Superscript III Rnase and the quantification relied on fluorescent-based Realtime PCR quantification using the LightCycler 480 (Roche Diagnostics, Mannheim, DE), using the previously reported PCR conditions [21] and the primers listed in Table 2. Absolute quantification of mRNA copies was performed as published [22]. PCR amplification efficiency varied between 1.93 and 2.20. Normalization was performed using the following reference genes, as reported [23]: glucose-6-phosphate dehydrogenase (G6PD), peptidylprolyl isomerase B (cyclophilin B) (PPIB) and 18S ribosomal RNA (18S). Quantitative Real-Time PCR analyses were compliant with MIQE guidelines [24, 25]*.*

**Table 2:** Sequence primers and gene description

Gene Symbol	Description	GenBank	size (bp	Primer sequence 5′ → 3′ S/AS
SQSTM1	*Homo sapiens* sequestosome 1 (SQSTM1), region common to the 3 transcripts	NM_003900	157	GGCGGAGCAGATGAGGAAGAT/TGGCATCTGTAGGGACTGGAG
TM7SF4	Homo sapiens dendrocyte expressed seven transmembrane protein (DCSTAMP)	NM_030788	161	GCAACCTAAGGGCAAAGAGCT/ATGGCTGGGACTGAAAAGAGAGACT
OS9	Homo sapiens osteosarcoma amplified 9, endoplasmic reticulum lectin (OS9), 8 transcripts	NM_006812	162	CACCCTTCCCTACAGCCTGAG/GCTCGCACCTGCCATCTTTTG
CREB3	Homo sapiens cAMP responsive element binding protein 3	NM_006368	108	AAACGTGTGCGGAGGAAGATTC/GGCTGTGTATTTCAAGACCCTGCT
LAMP1	Homo sapiens lysosomal associated membrane protein 1 (LAMP1)	NM_005561	129	ATGGGGCTGCAGCTGAACCT/CAGCTCCAGAGTCACCAGGT
OC-STAMP	Homo sapiens osteoclast stimulatory transmembrane protein (OCSTAMP)	NM_080721	100	AGGAGGAGCTGTTGAGTTGTCTTC/AGGAGGAAGGCTACATGGTCTG
NFATc1	Homo sapiens nuclear factor of activated T-cells, cytoplasmic, calcineurin-dependent 1 (NFATC1),7 transcripts	NM_006162	204	AGCGAAAACTGACCGGGACCT/GGCTCATAATCATCAGTGGGTTCT
G6PD	Homo sapiens glucose-6-phosphate dehydrogenase (G6PD), nuclear gene encoding mitochondrial protein	NM_000402	121	GATGTCCCCTGTCCCACCAACTCTG/GCAGGGCATTGAGGTTGGGAG
PPIB	Homo sapiens peptidylprolyl isomerase B (cyclophilin B) (PPIB)	NM_000942	179	GAAGAAGGGGCCCAAAGTCAC/CACGATGGAATTTGCTGTTTTTGTAG
18S	Homo sapiens 18S ribosomal RNA	NR_003286	226	ACGGACCAGAGCGAAAGCATT/TCCGTCAATTCCTTTAAGTTTCAGCT
ADNg	Homo sapiens 3-beta-hydroxysteroid dehydrogenase/delta-5-delta-4-isomerase (3-beta-HSD) gene (intron)	M38180	260	GAAGGGCAGAGGTGGAACTAGAA/AACAAAGACCAAAGACCAGTGAGA

### Western blot analyses

Cell lysates were separated by SDS-PAGE, and transferred to a PVDF membrane. The membrane was incubated with the primary antibodies. We used a primary antibody against DC-STAMP, OC-STAMP, SQSTM1, LAMP1, OS-9 and CREB3 also known as Luman, and an anti-alpha tubulin or vinculin antibody as a loading control. HRP-conjugated secondary antibodies were used to achieve detection with chemiluminescent system. Protein expression was quantified by densitometry using the Molecular Imager Gel Doc XR and Imaging System.

### Statistical analyses

We compared osteoclast phenotype characteristics, gene and protein expressions in three groups consisting in PDB patients (with or without the rs62620995 rare variant) and from healthy donors non carrier of this variant. Statistical analyses, performed by the use of GraphPadPrism, relied on ANOVA followed by Tukey post-tests. *P*-values <0.05 were considered statistically significant.

## Results

### Osteoclast morphology and bone resorption abilities

**Fig. 1:**
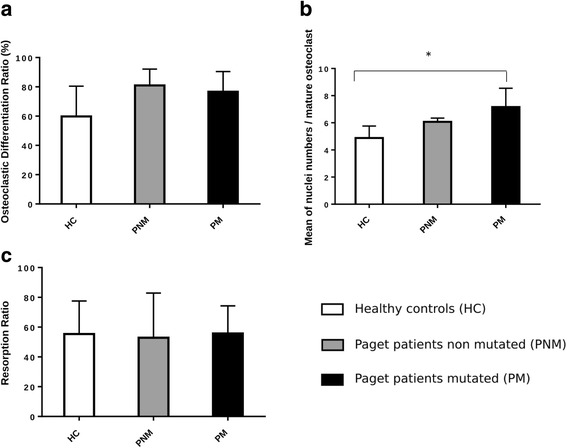
In vitro differentiated osteoclast morphology and bone resorption abilities: **a** Percentage of multinucleated cells (MNC), defined by three nuclei or more per cell, per total number of cells with an actin ring; **b** Number of nuclei per MNC on 20 cells randomly selected per well; **c** Bone resorption abilities presented as the percentage of bone resorbed area; * *p* < 0.05

At the end of the differentiation period (21 days), the percentage of multinucleated cells was higher, but not statistically significant, both in patients non carrier of the variant (80.9 ± 12.8%) and carrier of the variant (76.6 ± 26.8%), than in healthy controls (59.7 ± 23.8%). The mean number of nuclei per multinucleated cell was significantly higher in patients carrier of the variant (7.2 ± 4.3) than in healthy controls (4.9 ± 2.0), *p* = 0.04. But this mean number of nuclei in patients carrier of the variant was not significantly different from patients non carrier of the variant (6.1 ± 2.9). The mean area of resorbed bone did not differ significantly when comparing the three groups (Fig. 1). The ratio of the mean area of resorbed bone by the percentage of multinucleated cells was 0.93 in healthy controls, 0.73 in patients carrier of the variant and 0.65 in patients non carrier of the variant. Raw data of osteoclast phenotype are available in Additional file 1.

### Immunofluorescence of the DC-STAMP protein

**Fig. 2:**
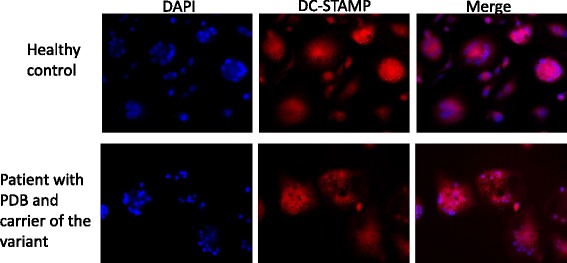
Immunofluorescence of DC-STAMP in osteoclast cultures at day 21 in cells from a healthy donor and a patient with Paget’s disease of bone (PDB) and carrier of the rare variant

The immunofluorescence of the DC-STAMP protein in osteoclast cultures after 21 days of differentiation showed a similar distribution between the patient with PDB carrier of the rare variant and a healthy control, mainly intracellular internalized (Fig. 2).

### Gene expression analyses from cell lysates of in vitro differentiated osteoclasts

**Fig. 3:**
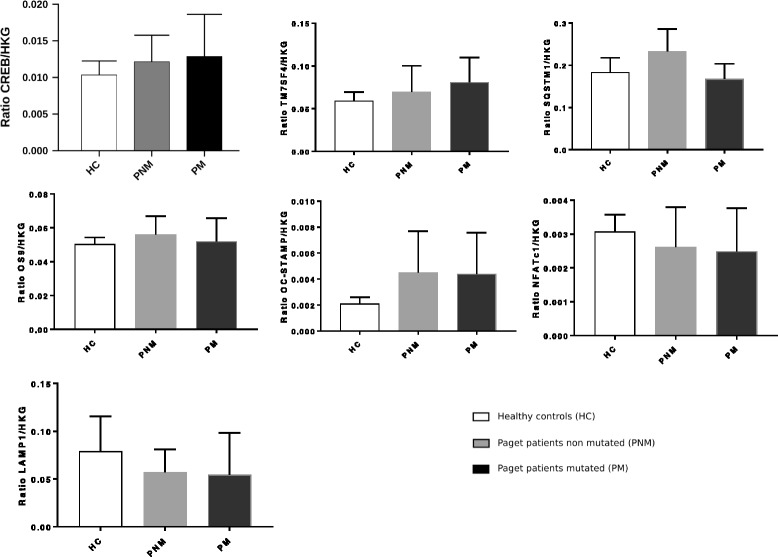
Gene expression analyses of candidate genes quantified by PCR from cell lysates of in vitro differentiated osteoclasts and normalized by the geometric mean of the three housekeeping genes. Footnote: The ratio corresponds to the ratio of the studied gene expression by the geometric mean of the gene expression of the three housekeeping genes

At the end of the differentiation period, gene expression analyses performed in cell lysates from mature osteoclasts and normalized by the geometric mean of the three housekeeping genes did not provide any significant differences between patients with PDB carrier of the rare variant, patients non carrier of the variant and healthy controls (Fig. 3, raw data available in Additional file 2). The gene expression analyses normalized by each housekeeping gene separately shown similar results (see Additional files 3, 4 and 5). In Fig. 3, gene expressions of *LAMP1* and *NFATc1* trended to be lower in patients, carrier of the variant or not, than in healthy controls, whereas gene expressions of *CREB*, *TM7SF4* and *OC-STAMP* trended to be higher in patients than in healthy controls.

### Protein expression analysis by western blot in cell lysates of in vitro differentiated osteoclasts

**Fig. 4:**
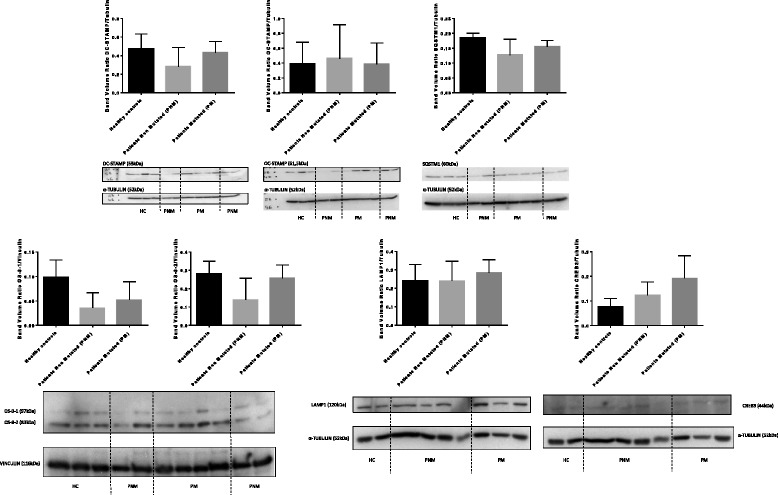
Protein expression analyses quantified by Western blot from cell lysates of in vitro differentiated osteoclasts

Western-Blot analyses in cell lysates of in vitro differentiated osteoclasts after 21 days of differentiation provided no statistically significant differences when comparing patients with PDB carrier of the rare variant, patients non carrier of the variant and healthy controls (Fig. 4, raw data available in Additional file 6).

In comparison to healthy controls, DC-STAMP expression was lower in patients non carrier of the variant but not in patients carrier of this rare variant. SQSTM1 and OS-9 expressions were lower in patients than in healthy controls, in particular in patients non carrier of the rare variant for isoforms 1 and 2 of OS-9 which were more than twice lower than in healthy controls (see the left part of the bottom line of Fig. 4). Patients carrier of the rare variant had the highest mean level of expression of CREB3 in comparison to patients non carrier of the variant and healthy controls.

## Discussion

In this study, we investigated the functional effects of a rare genetic variant, rs62620995, within *TM7SF4* gene at locus *8q22*, on osteoclast phenotype, as well as on gene and protein expressions in in vitro differentiated osteoclasts from patients with PDB, carrier of this rare variant or not, and healthy controls. We found that osteoclasts differentiated in vitro for 21 days from patients carrier of this variant significantly yielded to a higher number of nuclei per cell in comparison to healthy controls, suggesting a faster cell fusion in pagetic patients carrier of the variant. Although gene expression analyses and Western Blots did not provide any significant results, we observed that DC-STAMP expression was similar in patients carrier of the variant than in healthy controls which was unexpected at the end of the differentiation period of 21 days. Indeed, DC-STAMP, like other fusion regulators such as DAP12/FcRγ, TRAF6, NFATc1, MITF, CD47/TSP1, ATP6v0d2, CD44 and ADAM8/α_9_β_1_-integrin, is mainly expressed in committed pre-osteoclast as a critical player for fusion into multinucleated osteoclast [26]. But after the cell fusion which is known to occur earlier during the osteoclastogenesis of pagetic osteoclasts than in healthy osteoclasts, DC-STAMP expression is expected to rapidly decrease. The persistence of a relative high expression of DC-STAMP in osteoclasts from patients carrier of the variant may suggest a possible effect of the rare variant on DC-STAMP protein stability. The overexpression of CREB3, also known as Luman, in osteoclasts from patients carrier of the variant is interesting since this transcription factor is known to regulate the multinucleation of osteoclasts by promoting mononuclear osteoclasts fusion through DC-STAMP induction and intracellular distribution during osteoclastogenesis [27]. Our results of immunofluorescence on mature osteoclasts shown intracellular internalized localization of DC-STAMP which was already reported in a fraction of RANKL-induced osteoclast precursors whereas a localization to the cell surface was also detected in osteoclasts and undifferentiated monocytes [28]. DC-STAMP is known to interact via the C-terminus cytoplasmic tail with both isoforms of OS-9, which is one of lectin that regulates endoplasmic reticulum-associated degradation and quality control of protein folding [29, 30]. This interaction with OS-9 isoform 1 results in a redistribution of DC-STAMP to the endoplasmic reticulum and Golgi intermediate compartment.

According to the literature, the osteoclast phenotype in PDB was reported to have several characteristics such as an increased osteoclast number, an increased number of nuclei per osteoclast, an increased bone resorption capacity per osteoclast, an increased responsitivity to 1,25-(OH)_2_ vitamin D3, RANKL and TNF, an increased expression of TAF12 and an increased production of IL6 per osteoclast [2]. To date, no genetic variant linked to PDB gave rise to the full osteoclast phenotype, as cited above. In vitro, the *p.Pro392Leu* mutation within *SQSTM1* gene, which is the most frequent mutation found in patients with PDB, transfected in osteoclast precursors lead to hyperresponsitivity to RANKL, TNF but not 1,25-(OH)_2_ vitamin D3. Although the number of osteoclast was increased in the mouse model expressing this mutation, the number of nuclei per osteoclast was not found to be increased [31, 32]. To date, the most complete pagetic phenotype of osteoclasts was reported in vitro and in vivo by the expression of the measles virus nucleocapsid gene [33].

DC-STAMP role in osteoclastogenesis and during the late stage of cell fusion has been intensively studied since the first publication of its role in osteoclast in 2004 [28]. A mouse model of DC-STAMP deficient mice was generated, displaying major defects in osteoclast multinucleation which reduce bone resorption, resulting in a phenotype of osteopetrosis. Although a common variant of *TM7SF4* gene was found to be associated to PDB in 2010 [9, 10], no functional studied of DC-STAMP in PDB have been published yet.

The small number of samples studied here, due to the rarity of this genetic variant in our French-Canadian cohort, may have limited the statistical power to demonstrate differences between groups. Moreover, we found that the global area of resorbed bone was similar in the three groups and the ratio of resorbed bone per percentage of multinucleated cell was the highest in healthy controls. This observation may be due to the concentration of 40 ng/ml of RANKL chosen for this project, which was enough for osteoclastogenesis but possibly not enough to stimulate pagetic osteoclast bone resorption. In accordance to the hyperresponsitivity to RANKL of pagetic osteoclasts, stimulation of bone resorption with higher concentration of RANKL up to 100 ng/ml was previously reported in in vitro differentiated human osteoclasts in PDB [34].

The functional effect of this rare genetic variant in *TM7SF4* gene should be further investigated in several ways, in PDB as well as in other bone disorders such as osteoporosis, giant cell tumors or osteopetrosis. DC-STAMP appears more and more in the literature as a possible novel therapeutic target for periodontal diseases [35], as a biomarker for psoriatic arthritis or as a way to deliver drugs in bone fractures sites [36]. In vitro studies should be undertaken to investigate the role of the rare genetic variant on the expression of DC-STAMP at different times during osteoclastogenesis and under different conditions such as with a gradient of concentration of RANKL or by the use of cocultures of osteoclast precursors with stromal cells to reproduce more physiological conditions like in the bone microenvironment. The folding of the protein and the impact of the rare variant, located in the seventh and last transmembrane domain of the protein, on the Immunoreceptor Tyrosine-based Inhibitory Motif (ITIM)-mediated signaling of the cytoplasmic tail of DC-STAMP should also be further studied [37].

## Conclusions

Our results suggest that the rare genetic variant of *TM7SF4* gene, found in our French-Canadian cohort of patients with PDB and which encodes the DC-STAMP protein, increase the number of nuclei per multinucleated cells and affect DC-STAMP expression during osteoclastogenesis in PDB. Further analyses will help at better understanding the role of this rare genetic variant on osteoclast morphology and osteoclastogenesis.

## Additional files

Additional file 1: Raw data of osteoclast phenotype consisting in number of multinucleated cells, number of nuclei per multinucleated cell and bone resorbed area. (DOC 25 kb)

Additional file 2: Raw data of quantitative PCR analyses. (DOC 45 kb)

Additional file 3: Gene expression analyses of candidate genes quantified by PCR from cell lysates of in vitro differentiated osteoclasts and normalized by G6PD. (PPT 240 kb)

Additional file 4: Gene expression analyses of candidate genes quantified by PCR from cell lysates of in vitro differentiated osteoclasts and normalized by PPIB. (PPT 244 kb)

Additional file 5: Gene expression analyses of candidate genes quantified by PCR from cell lysates of in vitro differentiated osteoclasts and normalized by 18S. (PPT 239 kb)

Additional file 6: Raw data of Western Blot analyses. (DOC 30 kb)
