# Supplementary figures and images for: Effect of a rare genetic variant of TM7SF4 gene on osteoclasts of patients with Paget’s disease of bone
Source: BMC Med Genet. 2017 Nov 16;18:133. doi: 10.1186/s12881-017-0495-3 (PMC5693359; doi:10.1186/s12881-017-0495-3)

## Slide 1
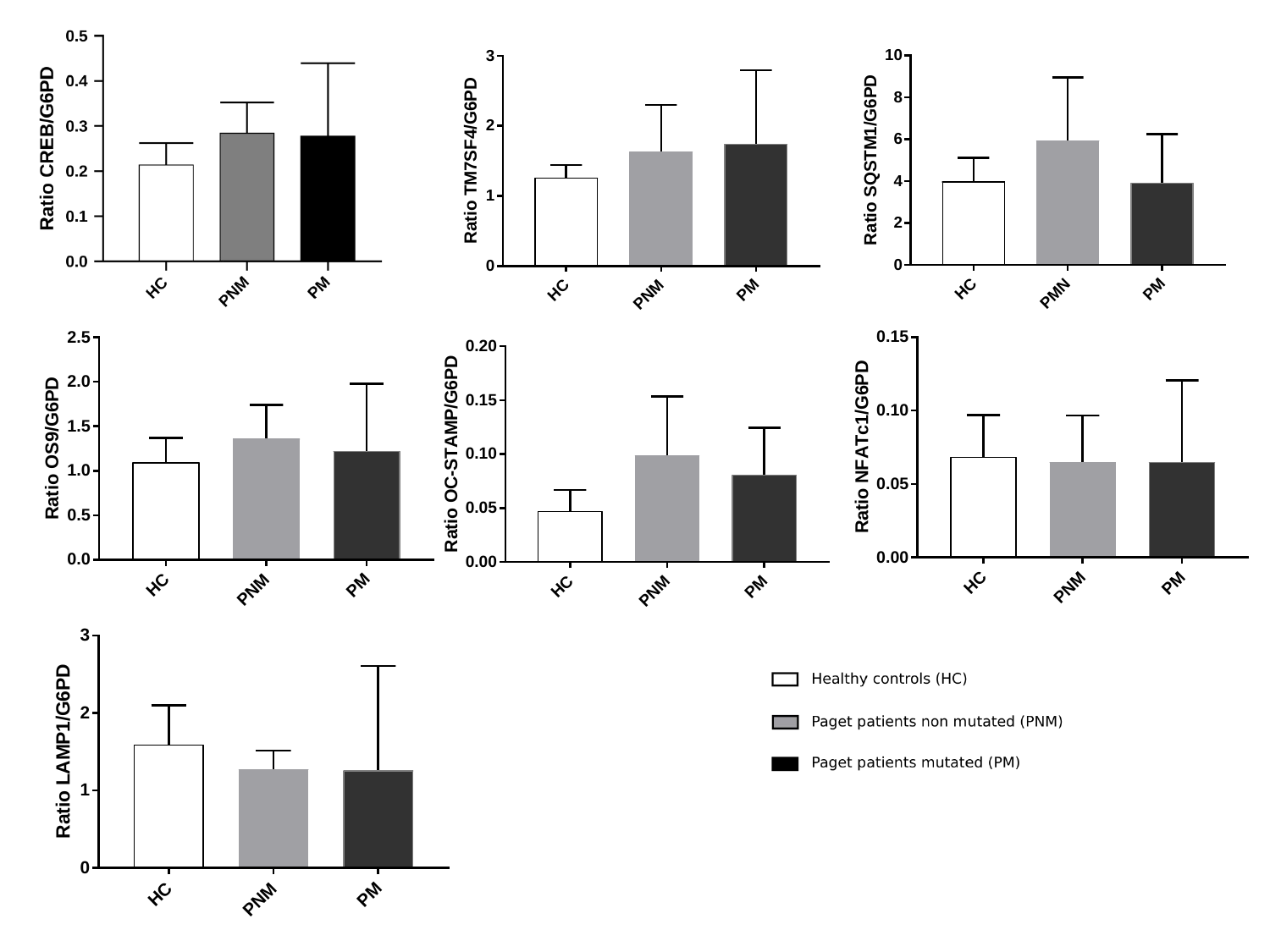

Supplement: Supplementary file 3 — Gene expression analyses of candidate genes quantified by PCR from cell lysates of in vitro differentiated osteoclasts and normalized by G6PD. (PPT 240 kb) [file 12881_2017_495_MOESM3_ESM.ppt]

## Slide 1
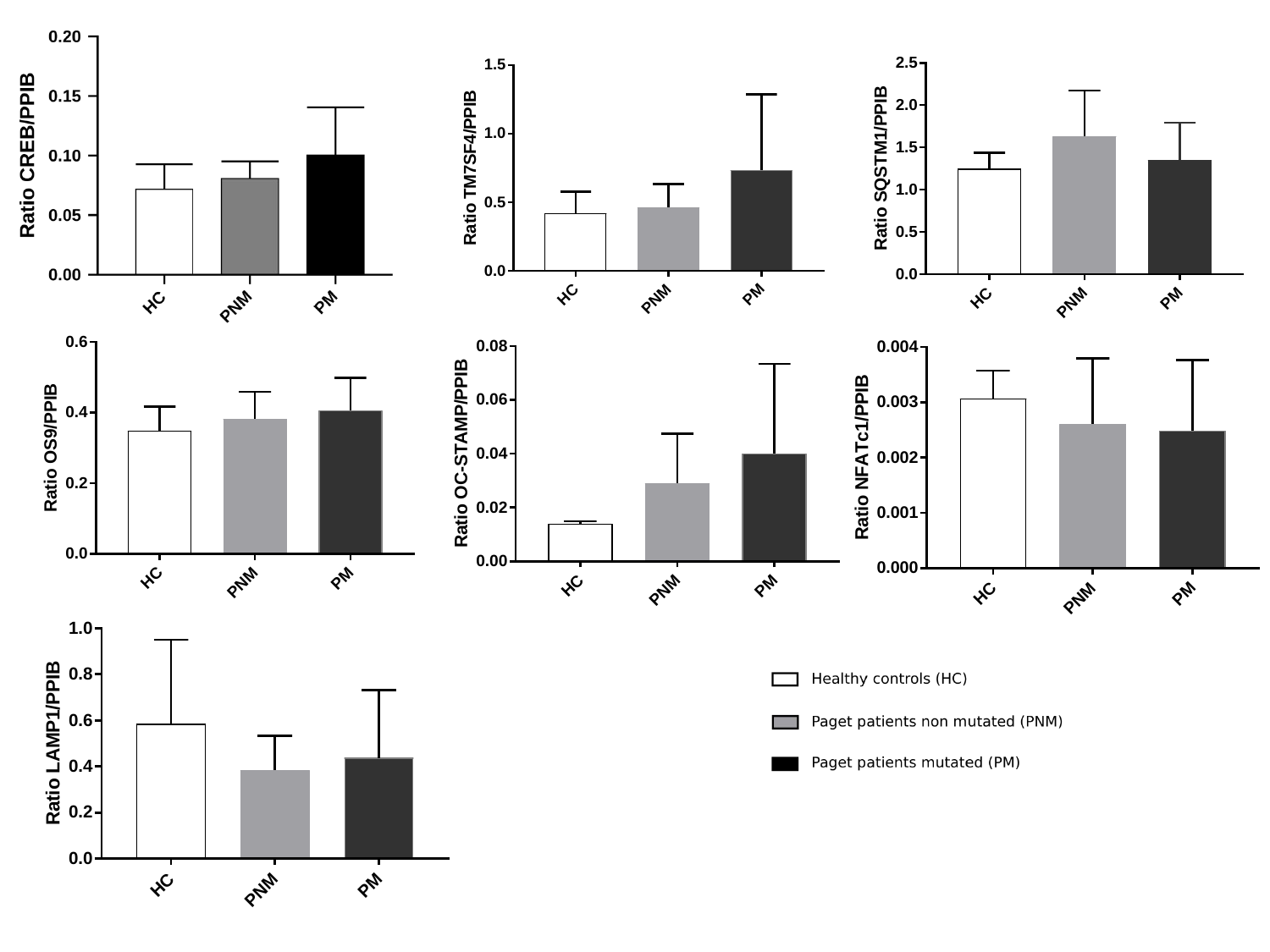

Supplement: Supplementary file 4 — Gene expression analyses of candidate genes quantified by PCR from cell lysates of in vitro differentiated osteoclasts and normalized by PPIB. (PPT 244 kb) [file 12881_2017_495_MOESM4_ESM.ppt]

## Slide 1
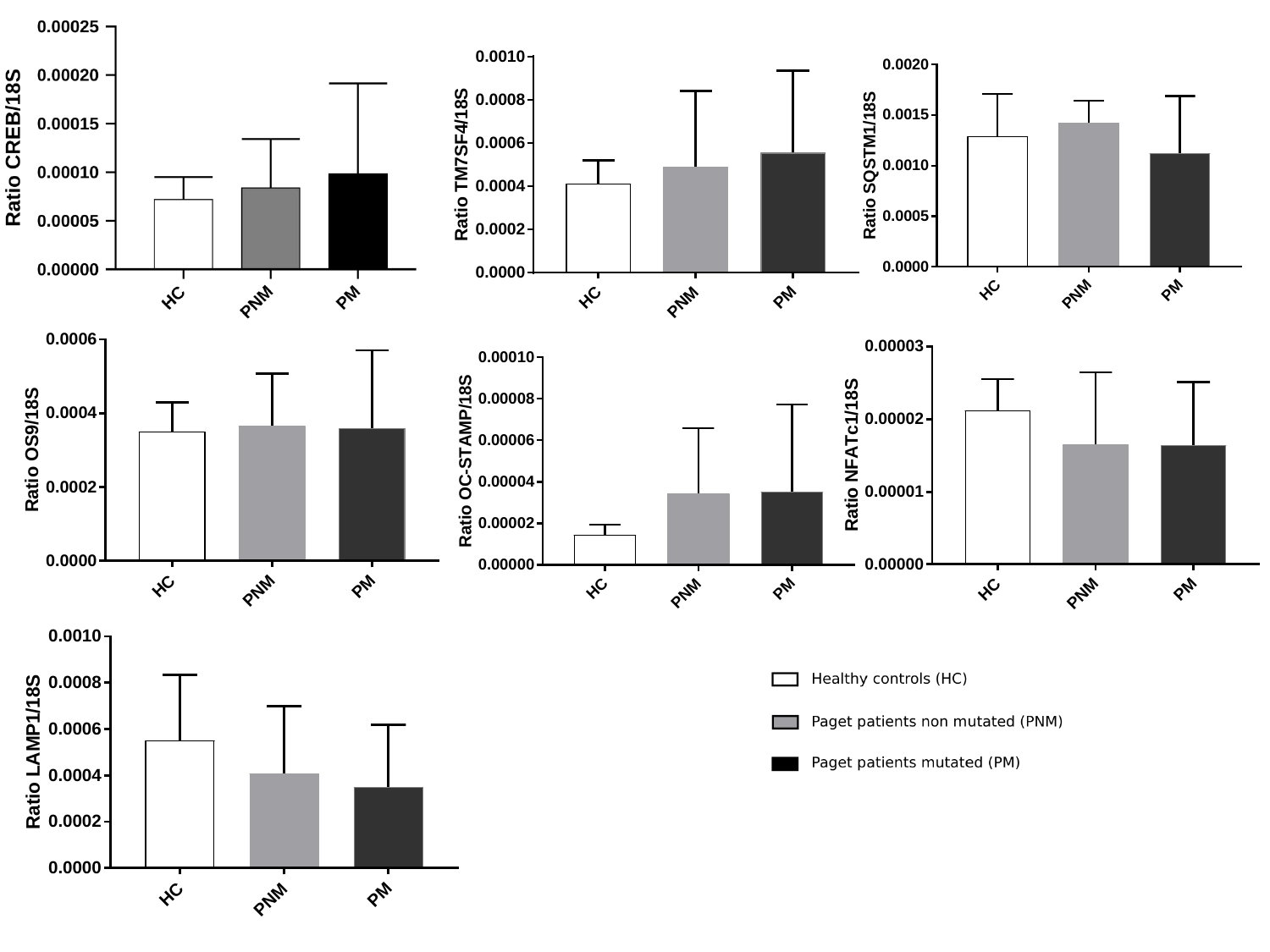

Supplement: Supplementary file 5 — Gene expression analyses of candidate genes quantified by PCR from cell lysates of in vitro differentiated osteoclasts and normalized by 18S. (PPT 239 kb) [file 12881_2017_495_MOESM5_ESM.ppt]
