# Supplementary material for: Effect of a rare genetic variant of TM7SF4 gene on osteoclasts of patients with Paget’s disease of bone
Source: BMC Med Genet. 2017 Nov 16;18:133. doi: 10.1186/s12881-017-0495-3 (PMC5693359; doi:10.1186/s12881-017-0495-3)
Supplement: Supplementary file 2 [file 12881_2017_495_MOESM1_ESM.doc]

Participant status	Percentage of multinucleated cells	Mean number of nuclei per multinucleated cell	Percentage of bone resoprtion
Healthy control	74.65	6.17	62.499
Healthy control	75.55	6.20	63.708
Healthy control	69.08	5.89	22.915
Healthy control	74.79	6.04	72.522
Patient with Paget's disease non carrier of the genetic variant	79.63	6.66	73.526
Patient with Paget's disease non carrier of the genetic variant			66.642
Patient with Paget's disease non carrier of the genetic variant	77.36	6.61	18.52
Patient with Paget's disease non carrier of the genetic variant	74.68	6.89	
Patient with Paget's disease carrier of the TM7SF4 genetic variant	71.34	6.61	34.177
Patient with Paget's disease carrier of the TM7SF4 genetic variant	74.75	8.10	67.879
Patient with Paget's disease carrier of the TM7SF4 genetic variant	59.63	8.00	64.943
Patient with Paget's disease carrier of the TM7SF4 genetic variant		7.21	
