# Supplementary material for: Effect of a rare genetic variant of TM7SF4 gene on osteoclasts of patients with Paget’s disease of bone
Source: BMC Med Genet. 2017 Nov 16;18:133. doi: 10.1186/s12881-017-0495-3 (PMC5693359; doi:10.1186/s12881-017-0495-3)
Supplement: Supplementary file 3 [file 12881_2017_495_MOESM2_ESM.doc]

	Gene Symbol	Mean gene expression	Mean G6PD expression	Mean PPIB expression	Mean 18S expression	Geometric mean of the three housekeepng genes
Paget Patients carrier of the variant	**SQSTM1**	5056611,142	2480257,621	2661253,2	10260509726	40707804,25
Healthy control	**SQSTM1**	4721777,451	1161497,103	3420961,177	2839062899	22348366,47
Paget Patients carrier of the variant	**SQSTM1**	4348489,831	742100,7082	3040987,161	3818008896	20498779,32
Healthy control	**SQSTM1**	3976193,553	1648722,862	2940804,354	4799492805	28476753,66
Healthy control	**SQSTM1**	4911330,445	1131178,133	3787513,595	3029518293	23426641,44
Paget Patients non carrier	**SQSTM1**	3861011,302	1267317,966	3420651,181	2352153210	21681543,57
Paget Patients non carrier	**SQSTM1**	7795720,317	779243,3006	3514007,178	6811226115	26474003,46
Healthy control	**SQSTM1**	4348712,747	849278,6089	4480461,829	4153010354	25086028,52
Paget Patients non carrier	**SQSTM1**	7255589,072	1161339,706	3703432,713	5256111446	28222307,97
Paget Patients non carrier	**SQSTM1**	3976295,468	870544,9873	3274115,35	2575117734	19424072,49
Paget Patients carrier of the variant	**SQSTM1**	4180659,693	2284025,595	3352113,532	2243126593	25799602,24
Paget Patients carrier of the variant	**SQSTM1**	3667200,51	608454,414	4344683,691	3679092160	21343654,51
Paget Patients carrier of the variant	**TM7SF4**	4010984,462	2480257,621	2661253,2	10260509726	40707804,25
Healthy control	**TM7SF4**	1585607,315	1161497,103	3420961,177	2839062899	22348366,47
Paget Patients carrier of the variant	**TM7SF4**	771778,2074	742100,7082	3040987,161	3818008896	20498779,32
Healthy control	**TM7SF4**	1833314,798	1648722,862	2940804,354	4799492805	28476753,66
Healthy control	**TM7SF4**	1232345,746	1131178,133	3787513,595	3029518293	23426641,44
Paget Patients non carrier	**TM7SF4**	2127347,714	1267317,966	3420651,181	2352153210	21681543,57
Paget Patients non carrier	**TM7SF4**	1744144,465	779243,3006	3514007,178	6811226115	26474003,46
Healthy control	**TM7SF4**	1245269,047	849278,6089	4480461,829	4153010354	25086028,52
Paget Patients non carrier	**TM7SF4**	823127,9464	1161339,706	3703432,713	5256111446	28222307,97
Paget Patients non carrier	**TM7SF4**	1678284,368	870544,9873	3274115,35	2575117734	19424072,49
Paget Patients carrier of the variant	**TM7SF4**	2437521,102	2284025,595	3352113,532	2243126593	25799602,24
Paget Patients carrier of the variant	**TM7SF4**	1988763,502	608454,414	4344683,691	3679092160	21343654,51
Paget Patients carrier of the variant	**OS9**	1310948,783	2480257,621	2661253,2	10260509726	40707804,25
Healthy control	**OS9**	1212392,429	1161497,103	3420961,177	2839062899	22348366,47
Paget Patients carrier of the variant	**OS9**	1310948,783	742100,7082	3040987,161	3818008896	20498779,32
Healthy control	**OS9**	1292361,984	1648722,862	2940804,354	4799492805	28476753,66
Healthy control	**OS9**	1233920,691	1131178,133	3787513,595	3029518293	23426641,44
Paget Patients non carrier	**OS9**	1010695,842	1267317,966	3420651,181	2352153210	21681543,57
Paget Patients non carrier	**OS9**	1256071,266	779243,3006	3514007,178	6811226115	26474003,46
Healthy control	**OS9**	1238420,326	849278,6089	4480461,829	4153010354	25086028,52
Paget Patients non carrier	**OS9**	1763928,365	1161339,706	3703432,713	5256111446	28222307,97
Paget Patients non carrier	**OS9**	1321149,937	870544,9873	3274115,35	2575117734	19424072,49
Paget Patients carrier of the variant	**OS9**	1434030,748	2284025,595	3352113,532	2243126593	25799602,24
Paget Patients carrier of the variant	**OS9**	1204043,093	608454,414	4344683,691	3679092160	21343654,51
Paget Patients carrier of the variant	**CREB3**	304833,8983	2480257,621	2661253,2	10260509726	40707804,25
Healthy control	**CREB3**	237936,4243	1161497,103	3420961,177	2839062899	22348366,47
Paget Patients carrier of the variant	**CREB3**	193513,2945	742100,7082	3040987,161	3818008896	20498779,32
Healthy control	**CREB3**	278686,459	1648722,862	2940804,354	4799492805	28476753,66
Healthy control	**CREB3**	299438,3241	1131178,133	3787513,595	3029518293	23426641,44
Paget Patients non carrier	**CREB3**	317245,1458	1267317,966	3420651,181	2352153210	21681543,57
Paget Patients non carrier	**CREB3**	255673,6953	779243,3006	3514007,178	6811226115	26474003,46
Healthy control	**CREB3**	205512,281	849278,6089	4480461,829	4153010354	25086028,52
Paget Patients non carrier	**CREB3**	246636,1252	1161339,706	3703432,713	5256111446	28222307,97
Paget Patients non carrier	**CREB3**	308260,8554	870544,9873	3274115,35	2575117734	19424072,49
Paget Patients carrier of the variant	**CREB3**	524934,3894	2284025,595	3352113,532	2243126593	25799602,24
Paget Patients carrier of the variant	**CREB3**	305960,131	608454,414	4344683,691	3679092160	21343654,51
Paget Patients carrier of the variant	**LAMP1**	1344182,409	2480257,621	2661253,2	10260509726	40707804,25
Healthy control	**LAMP1**	2556669,535	1161497,103	3420961,177	2839062899	22348366,47
Paget Patients carrier of the variant	**LAMP1**	2429313,521	742100,7082	3040987,161	3818008896	20498779,32
Healthy control	**LAMP1**	2994583,118	1648722,862	2940804,354	4799492805	28476753,66
Healthy control	**LAMP1**	1355084,332	1131178,133	3787513,595	3029518293	23426641,44
Paget Patients non carrier	**LAMP1**	1894829,308	1267317,966	3420651,181	2352153210	21681543,57
Paget Patients non carrier	**LAMP1**	779966,5685	779243,3006	3514007,178	6811226115	26474003,46
Healthy control	**LAMP1**	976323,0265	849278,6089	4480461,829	4153010354	25086028,52
Paget Patients non carrier	**LAMP1**	1683680,358	1161339,706	3703432,713	5256111446	28222307,97
Paget Patients non carrier	**LAMP1**	1012913,952	870544,9873	3274115,35	2575117734	19424072,49
Paget Patients carrier of the variant	**LAMP1**	1170616,504	2284025,595	3352113,532	2243126593	25799602,24
Paget Patients carrier of the variant	**LAMP1**	431972,4353	608454,414	4344683,691	3679092160	21343654,51

Paget Patients carrier of the variant	**OC-STAM**	191446,8398	2480257,621	2661253,2	10260509726	40707804,25
Healthy control	**OC-STAM**	48506,17592	1161497,103	3420961,177	2839062899	22348366,47
Paget Patients carrier of the variant	**OC-STAM**	16918,86383	742100,7082	3040987,161	3818008896	20498779,32
Healthy control	**OC-STAM**	39503,07813	1648722,862	2940804,354	4799492805	28476753,66
Healthy control	**OC-STAM**	57048,33115	1131178,133	3787513,595	3029518293	23426641,44
Paget Patients non carrier	**OC-STAM**	166436,6002	1267317,966	3420651,181	2352153210	21681543,57
Paget Patients non carrier	**OC-STAM**	69712,28893	779243,3006	3514007,178	6811226115	26474003,46
Healthy control	**OC-STAM**	61103,19187	849278,6089	4480461,829	4153010354	25086028,52
Paget Patients non carrier	**OC-STAM**	30573,72034	1161339,706	3703432,713	5256111446	28222307,97
Paget Patients non carrier	**OC-STAM**	129609,7458	870544,9873	3274115,35	2575117734	19424072,49
Paget Patients carrier of the variant	**OC-STAM**	218363,7103	2284025,595	3352113,532	2243126593	25799602,24
Paget Patients carrier of the variant	**OC-STAM**	77333,71214	608454,414	4344683,691	3679092160	21343654,51
Paget Patients carrier of the variant	**NFATc1**	37956,11464	2480257,621	2661253,2	10260509726	40707804,25
Healthy control	**NFATc1**	71723,54633	1161497,103	3420961,177	2839062899	22348366,47
Paget Patients carrier of the variant	**NFATc1**	68519,85257	742100,7082	3040987,161	3818008896	20498779,32
Healthy control	**NFATc1**	72004,2416	1648722,862	2940804,354	4799492805	28476753,66
Healthy control	**NFATc1**	67102,58244	1131178,133	3787513,595	3029518293	23426641,44
Paget Patients non carrier	**NFATc1**	26706,50516	1267317,966	3420651,181	2352153210	21681543,57
Paget Patients non carrier	**NFATc1**	64508,16489	779243,3006	3514007,178	6811226115	26474003,46
Healthy control	**NFATc1**	92713,61018	849278,6089	4480461,829	4153010354	25086028,52
Paget Patients non carrier	**NFATc1**	75354,07953	1161339,706	3703432,713	5256111446	28222307,97
Paget Patients non carrier	**NFATc1**	80001,5549	870544,9873	3274115,35	2575117734	19424072,49
Paget Patients carrier of the variant	**NFATc1**	50682,07801	2284025,595	3352113,532	2243126593	25799602,24
Paget Patients carrier of the variant	**NFATc1**	78991,44709	608454,414	4344683,691	3679092160	21343654,51
