# Supplementary material for: Effect of a rare genetic variant of TM7SF4 gene on osteoclasts of patients with Paget’s disease of bone
Source: BMC Med Genet. 2017 Nov 16;18:133. doi: 10.1186/s12881-017-0495-3 (PMC5693359; doi:10.1186/s12881-017-0495-3)
Supplement: Supplementary file 4 [file 12881_2017_495_MOESM6_ESM.doc]

Participant status	DC-STAMP	DC-STAMP/tubulin	OC-STAMP	OC-STAMP/Tubulin	SQSTM1	SQSTM1/tubulin	OS-9-1	OS-9-2	OS-9/vinculin	LAMP1	LAMP1/tubulin	CREB	REB/tubuli
Healthy control	4 697 136	13 887 180	2 130 551	9 039 870	2 378 651	12 233 795	511 296	2 777 760	8 772 576				
Healthy control	4 133 766	6 372 828	3 971 000	5 466 447	1 658 784	8 510 290	834 250	1 503 567	7 351 218	1 468 944	4 843 524	440 440	4 417 348
Healthy control	3 218 460	7 488 090	1 300 530	6 071 635	1 289 013	7 636 198	856 152	2 243 520	6 904 144	969 920	5 522 700	216 075	4 107 200
Paget's disease not carrier of the variant	401 276	6 755 613	52 416	7 015 669	507 416	7 673 575	9 525	726 525	7 998 102	2 080 520	11 248 009	652 982	8 488 576
Paget's disease not carrier of the variant	2 276 586	6 246 396	1 037 168	7 847 273	1 773 387	9 075 552	405 900	2 447 372	7 955 972	917 745	6 287 410	533 478	4 375 324
Paget's disease not carrier of the variant	3 709 910	7 058 478	3 946 908	4 501 575	656 600	5 726 400	426 756	740 850	6 002 892	1 078 402	4 540 349	666 450	3 342 647
Patient with Paget's disease not carrier of the variant	1 554 690	8 842 770	3 004 800	3 643 101	960 646	7 309 056	167 168	318 784	8 918 544	1 910 902	4 899 910	444 825	4 623 004
Patient with Paget's disease carrier of the variant	5 970 468	10 984 416	401 610	6 368 901	1 392 314	10 317 890	360 360	1 542 744	8 107 764				
Patient with Paget's disease carrier of the variant	2 480 028	6 931 155	2 939 943	6 273 705	1 014 020	7 182 546	215 775	2 181 000	6 878 775	1 276 870	6 286 596	402 670	4 716 800
Patient with Paget's disease carrier of the variant	2 125 662	7 146 755	3 879 116	5 289 193	1 065 050	5 828 550	649 252	1 954 368	6 053 048	1 232 914	3 912 930	837 225	3 445 455
Patient with Paget's disease carrier of the variant	4 140 552	7 895 580	1 425 853	5 446 764	1 064 490	6 695 114	242 400	1 908 480	9 455 712	1 899 852	5 702 536	1 106 550	4 488 864
